# Net energy of grains for dairy goats differed with processing methods and grain types

**DOI:** 10.1186/s40104-024-01136-y

**Published:** 2025-01-05

**Authors:** Xiaodong Su, Lei Zhang, Yiyang Sun, Yanbo Wu, Jianrong Ren, Shengru Wu, Xinjian Lei, Jun Zhang, Dangdang Wang, Hao Ren, Junhu Yao

**Affiliations:** 1 College of Animal Science and Technology, Northwest A&F University, Yangling, Shaanxi 712100 China; 2 Xi’an Wellhope Feed Technology Co., Ltd., Xi’an, Shaanxi 710000 China

**Keywords:** Dairy goats, Energy metabolism, Grain types, Processing, Starch digestion

## Abstract

**Background:**

The diverse types and processing methods of grains intricately influence the sites and digestibility of starch digestion, thereby impacting energy utilization. This study aimed to explore the impact of grain variety and processing methods on the net energy (NE) in dairy goats, analyzing these effects at the level of nutrient digestion and metabolism.

**Methods:**

Eighteen castrated Guanzhong dairy goats (44.25 ± 3.59 kg BW) were randomly divided into 3 groups, each consisting of 6 replicates. The substitution method was employed to determine the NE values of the dry-rolled corn (DRC), dry-rolled wheat (DRW) or steam-flaked corn (SFC, 360 g/L). Briefly, two phases were performed. Throughout the basal phase, all goats were fed the same basal diet. In the substitution phase, 30% of the basal diet was replaced with DRC, DRW and SFC, respectively.

**Results:**

In this study, the NE values of the DRC, DRW and SFC were 7.65, 7.54 and 7.44 MJ/kg DM, respectively. Compared to the DRC group, the DRW group showed increased digestibility of starch and crude protein (CP). Similarly, the SFC group exhibited increased organic matter (OM) and starch digestibility and a trend towards higher dry matter (DM) digestibility, reduced fecal OM and starch content. Additionally, fecal volatile fatty acid (VFA) concentrations decreased in goats fed SFC. Correspondingly, digestible energy (DE) in the DRW and SFC groups tended to be higher than in the DRC group. DRW increased total VFA concentration compared to DRC, while SFC increased the proportion of propionate and decreased the acetate-to-propionate ratio in the rumen. Both the DRW and SFC diets elevated serum glucose levels. Furthermore, heat increment (HI) and gaseous energy (GasE) related to fermentation were significantly higher in the DRW and SFC groups compared to the DRC group.

**Conclusion:**

Our findings indicated that DRW and SFC increased rumen starch fermentation in goats, thereby improving total tract starch digestion and DE. However, DRW and SFC failed to improve NE value due to increased heat and gas energy production from fermentation. Therefore, excessively refined grains processing in the diet of dairy goats does not effectively improve energy efficiency.

**Supplementary Information:**

The online version contains supplementary material available at 10.1186/s40104-024-01136-y.

## Introduction

Grains are the primary source of energy in ruminant diets, with starch serving as the main energy component. The structure of the protein matrix in the endosperm varies among grain types and influences starch digestibility [1]. For instance, corn, which has a protein matrix predominantly composed of zein, ferments more slowly compared to wheat, whose protein matrix is mainly glutenin and ferments more rapidly [2]. Processing treatments like steam flaking can overcome the structural constraints to digestion in varying degrees, enhancing digestion of the grain [3]. Compared to dry-rolled corn (DRC), both steam-flaked corn (SFC) and dry-rolled wheat (DRW) significantly improve rumen and total starch digestibility [4–6], thereby increasing energy availability for dairy and beef production [7, 8]. However, a study on goats found that adding SFC to diet did not improve nutrient digestion or growth performance [9]. Furthermore, goats fed corn diets demonstrated superior growth compared to those on wheat diets [10]. SFC and DRW have undergone extensive research and have found widespread application in dairy and beef cattle diets. However, the knowledge of their effects on goat nutrition is incomplete. Thus, a thorough understanding of how different grain types and processing methods affect energy metabolism and their applications in goat feeding is still needed.

The energy content of a test feedstuff can be evaluated using either the direct or the indirect (substitution) method [11, 12]. The direct method involves formulating a diet where all components are supplied solely by the test feedstuff. However, many ingredients (e.g., grains) cannot be fed to ruminants alone. To address this, the substitution method is employed. The substitution method, a form of the indirect method, involves replacing a portion of the basal diet with the test feedstuff, and observing the resulting change in energy content [12]. The available energy values of the test ingredients are calculated according to the equations of the substitution method. The recommended substitution rate for energy feeds generally ranges from 20% to 30%, with several studies supporting a 30% replacement rate as optimal [13, 14]. Compared to digestible energy (DE) and metabolizable energy (ME) systems, the net energy (NE) system provides a more accurate reflection of the actual available energy that the feedstuff provides to the goat and reduces production costs. Indirect calorimetry, a method that measures gas exchanges, specifically respiratory oxygen and carbon dioxide, is a reliable technique for determining NE. It has been pivotal in quantifying the energy released as heat or heat production (HP) and has been employed in many studies [13, 15–17]. Accurate estimations of energy availability in animal feeds are essential for developing comprehensive systems to delineate nutrient requirements [18]. However, the available energy values of the 3 conventional grains, DRC, DRW and SFC, for goats remain inadequately defined.

Therefore, this study employed the substitution method to evaluate the available energy and the impacts on nutrient digestion and metabolism of 3 starch sources (DRC, RWC, and SFC) in dairy goats. The results will provide foundational data to enhance precision nutrition for dairy goats.

## Materials and methods

### Animals and experimental design

Eighteen healthy castrated Guanzhong male goats (44.25 ± 3.59 kg BW) were randomly divided into 3 groups, each consisting of 6 goats. The ingredients and nutrient levels of the diets are shown in Table 1. The substitution method was employed in this study. During the basal experimental phase, all goats were fed the same basal diet, and the available energy of the basal diet for each goat was determined. In the substitution experimental phase, the 3 groups were fed the test diets, which 30% of the basal diet was substituted with DRC, DRW, or SFC. SFC was prepared by conditioning the corn in a steam chamber at 105 °C for 40 min and then passing it through a roller. DRC and DRW were prepared by passing the grains through a roller mill. Both the basal and substitution experimental phases lasted 21 d each, with the initial 12 d allocated for diet acclimation and the remaining 9 d for sample collection and measurements. The SFC and DRC were sourced from the same batch of corn. The nutrient levels of the test ingredient are shown in Table 2. Each goat was housed individually in metabolic cages measuring 1.5 m × 1.0 m × 1.5 m and fed twice daily at 08:00 and 16:00 h. The animals were provided feed at a rate of 2.3% of BW per day (DM basis), and the goats had free access to drinking water. The amount of feed refused was recorded daily.

**Table 1:** Ingredients and nutrient levels of the experimental diets, % of DM

Item^1^	Basal diet	Test diets		
		DRC	DRW	SFC
Ingredient				
Corn silage	46.20	32.14	32.14	32.14
Alfalfa hay	23.80	16.56	16.56	16.56
Ground corn	15.00	10.44	10.44	10.44
Dry-rolled corn	-	30.00	-	-
Dry-rolled wheat	-	-	30.00	-
Steam-flaked corn	-	-	-	30.00
Wheat bran	5.95	4.14	4.14	4.14
Soybean meal	4.95	3.45	3.45	3.45
Rice bran meal	2.69	1.87	1.87	1.87
CaCO_3_	0.64	0.64	0.64	0.64
Salt	0.24	0.24	0.24	0.24
NaHCO_3_	0.24	0.24	0.24	0.24
CaHPO_4_	0.11	0.11	0.11	0.11
Premix^2^	0.18	0.18	0.18	0.18
Nutrient levels^3^				
DM	50.04	64.15	64.55	64.88
CP	11.48	10.64	11.25	10.49
Starch	17.59	34.59	32.82	35.89
NDF	36.00	28.33	28.68	26.90
ADF	21.65	15.73	16.02	15.55

*DRC* Dry-rolled corn, *DRW* Dry-rolled wheat, *SFC* Steam-flaked corn

^1^*DM* Dry matter, *CP* Crude protein, *NDF* Neutral detergent fiber, *ADF* Acid detergent fiber

^2^Premix (per kg) contains: Cu 370 mg, Fe 2,200 mg, Zn 1,800 mg, Mn 800 mg, I 30 mg, Se 30 mg, Co 50 mg, Vitamin A 200 kIU, Vitamin D_3_ 4,500 IU, Vitamin E 6,500 IU, Vitamin K_3_ 45 mg

^3^All nutrient levels were the measured values

**Table 2:** Nutrient levels of the test ingredients, % of DM

Item^1^	DRC	DRW	SFC
DM	92.84	92.61	93.37
CP	8.74	10.69	8.19
Starch	67.59	61.69	70.59
NDF	11.42	12.56	6.81
ADF	2.59	3.57	2.01
GE	16.15	16.85	16.07

*DRC* Dry-rolled corn, *DRW* Dry-rolled wheat, *SFC* Steam-flaked corn

All nutrient levels were the measured values

^1^*DM* Dry matter, *CP* Crude protein, *NDF* Neutral detergent fiber, *ADF* Acid detergent fiber, *GE* Gross energy

### Sample collection and measurement

For this study, four environmentally controlled indoor chambers were used. The dimensions of each chamber were 7.4 m × 4.2 m × 2.7 m. Two chambers were designated for adaptation, while the other two were used for gas measurement. The construction, operation and animal welfare considerations of environmentally controlled chambers have been described in detail by Li et al. [19]. The methane (CH_4_), carbon dioxide (CO_2_), and oxygen (O_2_) emissions from each goat were measured using the approach of Li et al. [19] and Goopy et al. [20]. By this approach, gas emissions from each goat were quantified by measuring the gas accumulation within individual airtight chambers of fixed volume over a defined time interval. To ensure proper mixing, the air inside each chamber was agitated for 30 s every 10 min using 4 draft fans. Gas emissions from each goat were measured in 2 consecutive days over 3 distinct time intervals: 08:00 to 12:00 h and 16:00 to 20:00 h on the first day, and 00:00 to 04:00 h on the second day. According to Goopy et al. [20], the daily (24-h) quantity of gas emissions is determined by multiplying the representative 12-h data by the factor of 24/12. Following the conclusion of each interval, the chamber doors were opened to facilitate air exchange, and thorough cleaning was conducted before the subsequent period. The methane (CH_4_), carbon dioxide (CO_2_), and oxygen (O_2_) contents were measured using a gas chromatograph (7890B, Agilent Technologies, CA, USA) equipped with the Thermal Conductivity Detector (TCD), Flame Ionization Detector (FID), HP-PLOT Al_2_O_3_S PT, and HyasSepQ + MolSieve 5A columns (Agilent Technologies, CA, USA).

During the sampling period, feed refusals and spillage were collected and recorded for 5 consecutive days, while total feces and urine were completely collected concurrently. Feces and urine were collected twice daily at 07:30 and 15:30 h; urine from each goat was collected from a plastic bucket containing 20 mL of 10% hydrochloric acid and filtered through cotton gauze. The total amount of feces and urine produced by each goat was measured and 20% of the daily fecal output and 10% of the urinary excretion were stored at −20 °C, respectively. Subsequently, all the feed refusals, feces and urine samples collected over 5 d from each goat were combined, thoroughly mixed and subsampled, respectively. Samples of the diets, feed refusals, and feces were dried at 65 °C for 72 h, followed by grinding through a 1-mm screen. These samples were analyzed for dry matter (DM, AOAC 2023, 930.15), ash (AOAC 2023, 942.05), and crude protein (CP, AOAC 2023, 984.13) [21]. Additionally, neutral detergent fiber (NDF) and acid detergent fiber (ADF) were analyzed using an Ankom A200I fiber analyzer (ANKOM Technology, Macedon, NY, USA) as described by Mertens [22] and AOAC (2023, 973.18) [21], respectively. The starch content was determined using commercial kits (Nanjing Jiancheng Bioengineering Institute, Nanjing, China) based on the anthrone method. The gross energy (GE) content was determined utilizing a bomb calorimeter (6100, Parr, Moline, IL, USA).

On the final day of the sampling period, blood, ruminal fluid, and rectal feces samples were collected. Blood samples were obtained before the morning feeding, and at the 2^nd^, 4^th^, and 6^th^ h post-feeding. These samples were drawn from the jugular vein into 5-mL vacuum blood collection tubes. Subsequently, the samples were placed in a 37 °C water bath for 30 min and centrifuged at 3,500 × *g* for 10 min to separate the serum. Serum biochemical indices, including glucose (GLU), triglyceride (TG), total cholesterol (CHO), albumin (ALB), total protein (TP), and urea, were analyzed using an automated chemistry analyzer (BK-400, Biobase, Shandong, China).

Ruminal fluid samples were collected at the 2^nd^, 4^th^, and 6^th^ h after feeding using an esophageal tube. Due to the limited amount of rumen fluid available before morning feeding, collection was not possible during this time. To minimize saliva contamination, the initial 30 mL of fluid was discarded, and approximately 40 mL of rumen fluid was collected. The samples were immediately filtered through quadruple-layer gauze, and the pH was measured. Furthermore, a 10-mL subsample was stored at −80 °C for later analysis of volatile fatty acid (VFA) and ammonia-nitrogen (NH_3_-N) concentrations. The VFA concentrations of each sample were analysed using a gas chromatograph (7890A, Agilent Technologies, CA, USA) equipped with a 30 m × 0.25 mm × 0.25 μm fused silica column (DB-FFAP, Agilent Technologies, CA, USA). Solid particles and proteins were removed from the samples prior to analysis, following the methods described by Li et al. [23]. The ruminal NH_3_-N concentration was determined using the colorimetric phenol-hypochlorite method described by Broderick and Kang [24].

For the analysis of fecal VFAs, samples were collected before morning feeding, as well as at 2^nd^ and 4^th^ h post-feeding. Due to the limited volume of samples at the 6^th^ h post-feeding, they were not collected. At each sampling time, an aliquot of each fecal sample was immediately stored at −80 °C. Approximately 1 g of each sample was mixed with 4 mL of water, stored at 4 °C for 24 h, and subjected to pH determination, VFAs extraction and analysis. For VFA determination, the mixed samples were centrifuged at 10,000 r/min for 10 min, and 2 mL of the supernatant were added with 500 µL of metaphosphoric acid (250 g/L) and stored at 4 °C for 4 h. Then the samples were centrifuged at 13,500 r/min for 15 min, and 1 mL of the supernatant was mixed with 200 µL of crotonic acid (2.45 g/L) and then filtered through a 0.45 µm filter.

### Calculation

Heat production (HP) was calculated by using the volumes of respiratory gas (O_2_, CO_2_, CH_4_) and urinary nitrogen (UN) excretion according to Brouwer [25]:

$$\mathrm{HP}\left(\mathrm{kJ}/\mathrm d\right)=16.18\times{\mathrm O}_2\left(\mathrm L/\mathrm d\right)+5.02\times{\mathrm{CO}}_2\left(\mathrm L/\mathrm d\right)-5.99\times\mathrm{UN}\left(\mathrm g/\mathrm d\right)-2.17\times{\mathrm{CH}}_4\left(\mathrm L/\mathrm d\right)$$

The digestible energy (DE), metabolizable energy (ME), gaseous energy (GasE), heat increment (HI), net energy for maintenance (NEm), fasting heat production (FHP), and net energy (NE) of the experimental diets were determined using the following equations:

$$\begin{aligned}\text{GasE }\left(\text{kJ}/\text{d}\right) &= 39.54 \times {\text{CH}}_{4} (\text{L}/\text{d})\\\text{DE }&=\text{ GE }-\text{FE}\\\text{ME }&=\text{ DE }-\text{ UE }-\text{ GasE}\\\text{HI }&=\text{ HP }-\text{ FHP}\\\text{NE }&=\text{ ME }-\text{ HI}\end{aligned}$$

where FE is the fecal energy, UE is the urinary energy and BW^0.75^ is the metabolic body weight. FHP is equivalent to the NE requirement for maintenance (NEm) [26].

$$\text{NEm }(\text{kJ}/\text{d}) = 315 \times {\text{BW}}^{0.75} (\text{kg})$$

The DE, ME, and NE contents of the test ingredients were calculated using the substitution method [11]:

$$\text{Eti }= [\text{Etd }- (1 -\text{ Rti}) \times \text{ Ebd}]/\text{Rti}$$

where Eti is the energy content of the test ingredient, Etd is the energy content of the test diet, Ebd is the energy content of the basal diet, and Rti is the ratio (i.e., 30%) of test ingredient substitution in the basal diet.

The apparent total tract digestibility (ATTD) of a nutrient was calculated using the following equation:

$$\text{ATTD }(\%) = (\text{NI }-{\text{NO}}_{\text{feces}})/\text{NI }\times 100$$

where NI is the nutrient intake, NO_feces_ is the nutrient output in feces.

### Statistical analysis

Outlying results were identified by Grubbs’ test. The data were analyzed by the General Linear Model (GLM) in SPSS 26 (SPSS INC., Chicago, USA) with diet and the test ingredient as a fixed effect and animal and chamber as random effects. The results are expressed as mean and standard error of the mean (SEM). The Duncan test was used to examine differences between treatment groups. *P* < 0.05 indicated statistical significance, and 0.05 < *P* < 0.10 indicated a trend toward statistical significance.

## Results

### Nutrient digestibility

The apparent nutrient digestibility results are presented in Table 3. Goats fed the SFC diet showed a significant increase in starch and OM digestibility (*P* < 0.05) and a tendency toward increased DM digestibility (*P* = 0.076) compared to those fed the DRC diet. Additionally, the DRW group exhibited significantly greater starch digestibility than those in the DRC group (*P* < 0.05). Furthermore, the CP digestibility was significantly greater in the DRW group compared to the other groups (*P* < 0.05). No significant differences were observed in the digestibility of NDF or ADF among the test diets (*P* > 0.05).

**Table 3:** Effects of different diets on DMI and apparent nutrient digestibility of the dairy goats

Item^1^	DRC	DRW	SFC	SEM	*P-*value
DMI, kg/d	1.04	1.07	1.03	0.035	0.866
Digestibility, % of DM					
DM	70.04	72.15	73.45	0.631	0.076
OM	72.00^b^	74.14^ab^	75.60^a^	0.628	0.050
CP	62.30^b^	68.97^a^	62.61^b^	1.089	0.009
Starch	91.93^b^	95.93^a^	96.63^a^	0.751	0.012
NDF	50.73	50.00	46.36	1.019	0.172
ADF	47.34	44.71	43.95	1.008	0.402

*DRC* Dry-rolled corn, *DRW* Dry-rolled wheat, *SFC* Steam-flaked corn, *SEM* Standard error mean

^1^*DMI* Dry matter intake, *DM* Dry matter, *OM* Organic material, *CP* Crude protein, *NDF* Neutral detergent fiber, *ADF* Acid detergent fibe

^a,b^ Means without a common superscript are significantly different from each other at *P* < 0.05 (*n* = 6)

### Ruminal fermentation characteristics

**Fig. 1:**
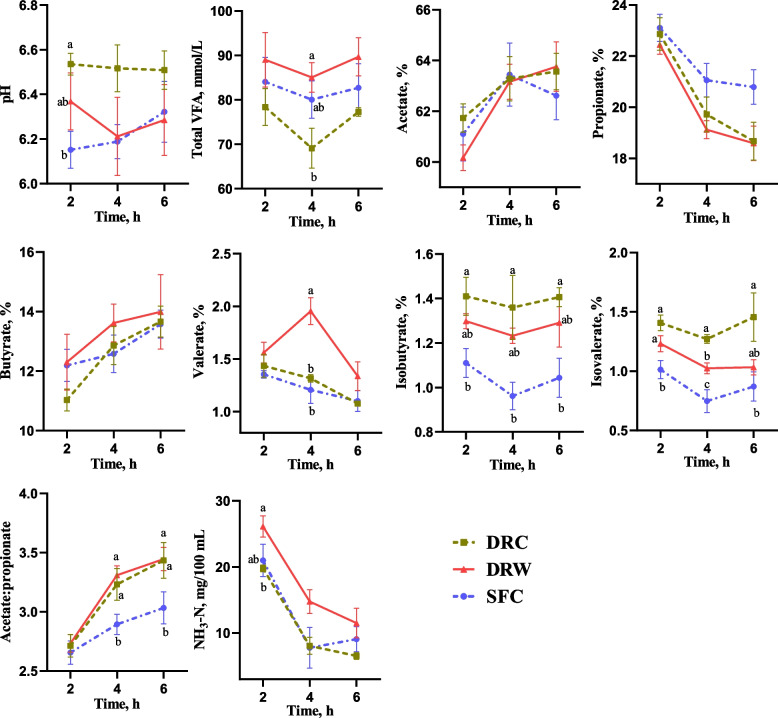
Effects of different diets on ruminal fermentation characteristics of the dairy goats. *DRC* Dry-rolled corn, *DRW* Dry-rolled wheat, *SFC* Steam-flaked corn. Time = hours after morning feeding. ^a–c^ Means without a common superscript are significantly different from each other at *P* < 0.05. *n* = 6

The ruminal fermentation characteristics are shown in Fig. 1. The ruminal pH of the SFC group was significantly lower than that of the DRC group at the 2^nd^ h post-feeding (*P* < 0.05). At the 4^th^ h, goats fed the DRW diet exhibited a significantly higher total VFA concentration compared to those fed the DRC diet (*P* < 0.05). Additionally, the SFC group tended to have an increased ruminal propionate molar proportion at the 6^th^ h after morning feeding (*P* = 0.070) and a significantly decreased acetate: propionate ratio at the 4^th^ and 6^th^ h (*P* < 0.05) compared to the DRC and DRW groups. However, the ruminal isobutyrate and isovalerate molar proportion were significantly lower in the SFC group from the 2^nd^ to 6^th^ h compared to the DRC group (*P* < 0.05). While the isovalerate molar proportion in the DRW group was also significantly lower than in the DRC group at the 4^th^ h (*P* < 0.05). Among goats fed the DRW diet, the valerate molar proportion was significantly greater than that in the other two groups at the 4^th^ h (*P* < 0.05), and the isovalerate molar proportion was significantly greater than that in the SFC group at the 2^nd^ and 4^th^ h (*P* < 0.05). The NH_3_-N concentration in the DRW group was significantly higher than that of the DRC group at the 2^nd^ h (*P* < 0.05).

Moreover, acetate molar concentration in the DRC group consistently remained lower than in the other two groups and was significantly lower than that of the DRW group at the 4^th^ h (*P* < 0.05, Fig. S1).

### Rectal fecal characteristics and nutrient concentration

**Fig. 2:**
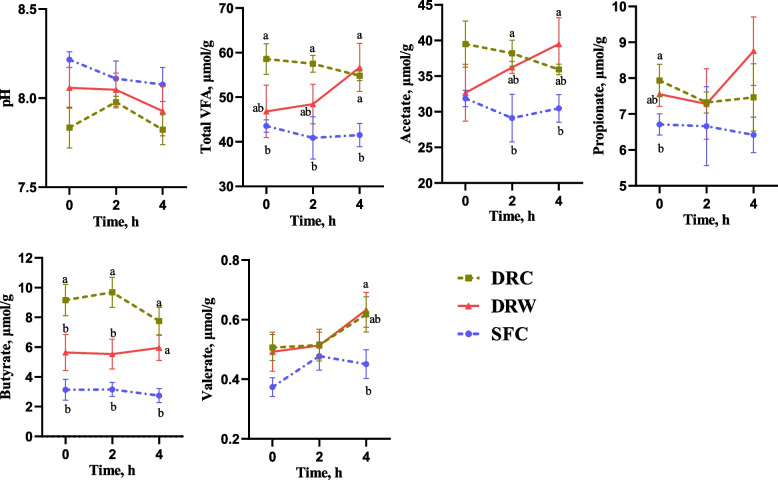
Effects of different diets on fecal characteristics of the dairy goats. *DRC* Dry-rolled corn, *DRW* Dry-rolled wheat, *SFC* Steam-flaked corn. Time = hours after morning feeding. ^a,b^ Means without a common superscript are significantly different from each other at *P* < 0.05. *n* = 6

The fecal fermentation characteristics are shown in Fig. 2. Compared to the DRC group, the SFC group showed a trend towards increased fecal pH before morning feeding (*P* = 0.063). Moreover, the SFC treatment significantly reduced the total VFA and butyrate concentrations from 0 to 4 h (*P* < 0.05). The propionate concentration in the SFC group was significant lower before morning feeding (*P* < 0.05), as well as the acetate concentration at the 2^nd^ h (*P* < 0.05) and the valerate concentration at the 4^th^ h (*P* < 0.05) compared to the DRC group. At the 4^th^ h, the total VFA, acetate, and butyrate concentrations in the SFC-fed goats were lower than those in the DRW group (*P* < 0.05). The butyrate concentration in the DRW group was lower than that in the DRC group from the 0 to 4 h (*P* < 0.05), with no significant difference from that of the SFC group (*P* > 0.05).

The fecal nutrient concentration is presented in Table 4. In the DRC group, the fecal OM concentration was significantly greater than that in the SFC group (*P* < 0.05), and the fecal starch concentration was significantly greater than that in both the DRW and SFC groups (*P* < 0.05). Conversely, the NDF and ADF concentrations were lower in the DRC-fed goats compared to those in the other 2 groups (*P* < 0.05).

**Table 4:** Effects of different diets on fecal nutrient content of the dairy goats, % of DM

Item^1^	DRC	DRW	SFC	SEM	*P*-value
OM	89.19^a^	88.65^ab^	87.84^b^	0.207	0.016
CP	13.34	13.26	12.47	0.185	0.100
Starch	9.22^a^	4.51^b^	4.45^b^	0.774	0.007
NDF	47.15^b^	54.10^a^	52.53^a^	0.936	< 0.001
ADF	27.85^b^	32.83^a^	31.52^a^	0.668	< 0.001

*DRC* Dry-rolled corn, *DRW* Dry-rolled wheat, *SFC* Steam-flaked corn, *SEM* Standard error mean

^1^*OM* Organic material, *CP* Crude protein, *NDF* Neutral detergent fiber, *ADF* Acid detergent fiber

^a,b^ Means without a common superscript are significantly different from each other at *P* < 0.05 (*n* = 6)

### Blood serum biochemical indices

**Fig. 3:**
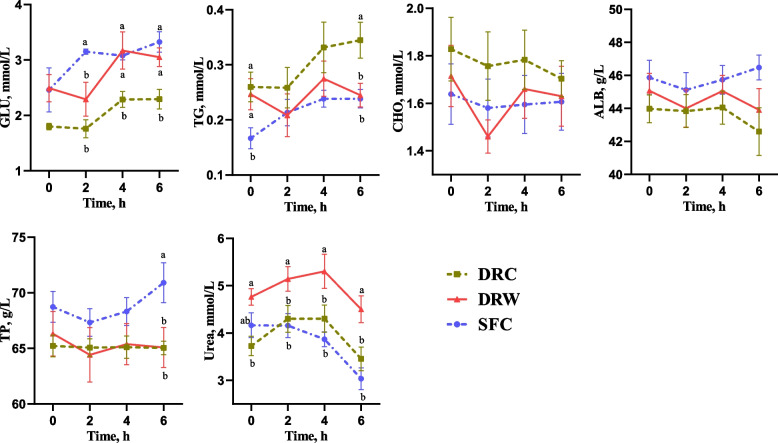
Effects of different diets on blood serum biochemical indices of the dairy goats. *DRC* Dry-rolled corn, *DRW* Dry-rolled wheat, *SFC* Steam-flaked corn. *GLU* Glucose, *TG* Triglyceride, *CHO* Total cholesterol, *ALB* Albumin, *TP* Total protein. Time = hours after morning feeding. ^a,b^ Means without a common superscript are significantly different from each other at *P* < 0.05. *n* = 6

As shown in Fig. 3, both the DRW and SFC groups exhibited significant greater serum GLU levels at the 4^th^ and 6^th^ h compared to the DRC group (*P* < 0.05). Additionally, at the 2^nd^ h, the SFC group exhibited significantly higher serum GLU levels than the other 2 groups (*P* < 0.05). Conversely, the serum TG levels were significantly lower in the SFC group before morning feeding and also lower than those in the DRC group at the 6^th^ h after feeding (*P* < 0.05). Similarly, the TG levels in the DRW group were lower than those in the DRC at the 6^th^ h (*P* < 0.05). Moreover, the serum TP levels in the SFC group were significantly greater than in the DRC and DRW groups at the 6^th^ h (*P* < 0.05). Furthermore, the serum urea levels in the DRW group were significantly greater than in the DRC group before morning feeding (*P* < 0.05) and consistently greater than in the other 2 groups from the 2^nd^ to 6^th^ h (*P* < 0.05). No significant difference in CHO concentration was found among the 3 groups (*P* > 0.05).

### Energy

**Fig. 4:**
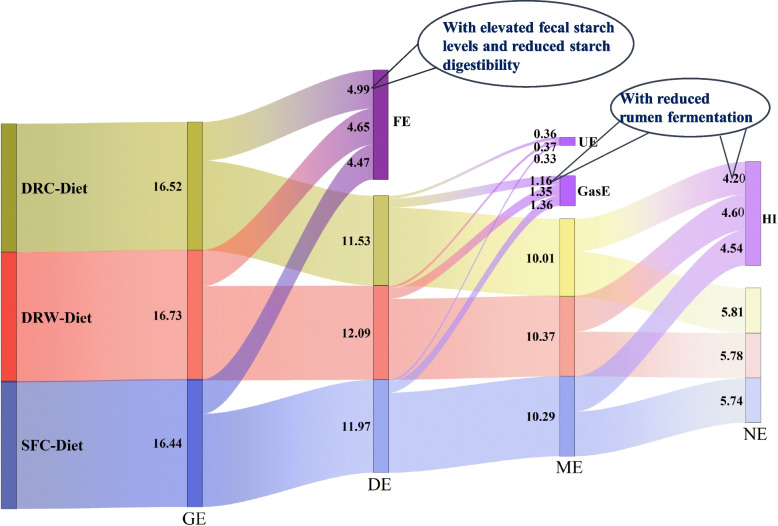
Energy distribution in different diets of the dairy goats (MJ/kg of DM). *DRC* Dry-rolled corn, *DRW* Dry-rolled wheat, *SFC* Steam-flaked corn. *GE* Gross energy, *FE* Fecal energy, *UE* Urinary energy, *GasE* Gaseous energy, *HP* Heat production, *HI* Heat increment, *DE* Digestible energy, *ME* Metabolizable energy, *NE* Net energy. *n* = 6

The energy data for the 3 experimental diets are presented in Table 5. The GE was significantly higher of the DRW group than that of the DRC and SFC groups (*P* < 0.05). And the GasE and HI of the DRC group were significantly lower than those of the other 2 groups (*P* < 0.05). Moreover, the DRC group showed a trend towards increasing excreted FE values (*P* = 0.089) while decreasing the DE values of the test diet and corn (*P* = 0.061, *P* = 0.062, respectively). In Table 6, the ME values of the 3 test ingredients, DRC, DRW, and SFC were 11.30, 12.42 and 12.15 MJ/kg DM, respectively. Correspondingly, the NE values were 7.65, 7.54, and 7.44 MJ/kg DM. However, no significant differences were observed in ME and NE values among the test diets or ingredients (*P* > 0.05). For a more intuitive visualization of energy distribution, we constructed an energy distribution Sankey diagram (Fig. 4). This diagram provides a clearer illustration of energy loss and transfer from the 3 diets across DE, ME, and NE levels.

**Table 5:** Effects of different diets on energy metabolism of the dairy goats, MJ/kg DM

Item^1^	DRC	DRW	SFC	SEM	*P-*value
Energy partition					
GE	16.52^b^	16.73^a^	16.44^b^	0.035	< 0.001
FE	4.99	4.65	4.47	0.100	0.089
UE	0.36	0.37	0.33	0.032	0.724
GasE	1.16^b^	1.35^a^	1.36^a^	0.036	0.025
HP	9.47	9.77	9.88	0.170	0.617
HI	4.20^b^	4.60^a^	4.54^a^	0.072	0.037
Available energy					
DE	11.53	12.09	11.97	0.105	0.061
ME	10.01	10.37	10.29	0.089	0.237
NE	5.81	5.78	5.74	0.105	0.968

*DRC* Dry-rolled corn, *DRW* Dry-rolled wheat, *SFC* Steam-flaked corn, *SEM* Standard error mean

^1^*GE* Gross energy, *FE* Fecal energy, *UE* Urinary energy, *GasE* Gaseous energy, *HP* Heat production, *HI* Heat increment, *DE* Digestible energy, *ME* Metabolizable energy, *NE* Net energy

^a,b^ Means without a common superscript are significantly different from each other at *P* < 0.05 (*n* = 6)

**Table 6:** The available energy of the 3 test ingredients, MJ/kg of DM

Item^1^	DRC	DRW	SFC	SEM	*P-*value
DE	12.86	14.61	14.26	0.329	0.062
ME	11.30	12.42	12.15	0.280	0.242
NE	7.65	7.54	7.44	0.327	0.969

*DRC* Dry-rolled corn, *DRW* Dry-rolled wheat, *SFC* Steam-flaked corn, *SEM* Standard error mean

^1^*DE* Digestible energy, *ME* Metabolizable energy, *NE* Net energy. *n* = 6

## Discussion

The type of cereal grain and the processing methods employed for corn grain influence digestive sites and starch digestion extent in ruminants, thereby impacting grain energy metabolism [5, 27]. The feed cost comprises a substantial proportion, ranging from 60% to 80%, of total livestock production costs. Therefore, any endeavor to reduce feed costs is expected to yield significant reductions in production costs and increase profits. In this study, the energy metabolism of goats fed DRC, DRW, and SFC test diets was systematically determined (Fig. 4). Additionally, the NE values of the tested ingredients were calculated. Specifically, the NE values of DRC, DRW, and SFC were determined as 7.65, 7.54, and 7.44 MJ/kg DM, respectively. Nutrient digestion, rumen and hindgut fermentation, and blood biochemistry were analyzed to elucidate energy metabolism at digestive and metabolic levels. The data obtained will serve as fundamental information for precision nutrition, reducing energy waste, and optimizing feed costs.

Our results indicated that, compared to the DRC group, the SFC group exhibited an increase in the starch and OM digestibility, with a tendency toward increased DM digestibility. The improved OM and DM digestibility in SFC-fed goats might primarily be attributed to the enhanced starch digestibility. These findings are in line with previous studies conducted on dairy cows [28–30]. This consistency may be due to the impact of steam flaking, which disrupts the protein matrix enveloping starch granules, subsequently enhancing starch gelatinization and increasing the surface area of corn [31]. Additionally, the larger particle size of SFC [28] enables longer retention in the rumen for enhanced digestion compared to DRC, thereby further contributing to increased starch digestibility. The starch digestibility of the DRW was also greater than that of the DRC group consistent with previous reviews by Huntington [6]. Compared to corn starch, wheat starch has a higher amylopectin content, increasing susceptibility to enzymatic hydrolysis [32]. Furthermore, the protein matrix surrounding wheat starch consists primarily of gluten, which is readily degraded in the rumen. Therefore, it does not impede microbial or enzymatic degradation of starch granules. In contrast, the protein matrix surrounding cornstarch granules is predominantly composed of prolamin zein, which is known for its insolubility in the ruminal environment [7]. Accordingly, the CP digestibility in the DRW group exceeded that in the other 2 groups. Additionally, the rumen NH_3_ concentrations and serum urea nitrogen levels were also elevated in the DRW group compared to the other 2 groups, aligning with a previous study conducted in dairy cows [33], which might be related to the urea nitrogen cycle in ruminants. In accordance with the nutrient digestibility data, the dietary and ingredient DE values were lower for goats fed the DRC diet.

The primary site for starch digestion typically occurs in the rumen [34], where microorganisms ferment starch into VFA. These VFAs are then absorbed by the host, where they play a crucial role in providing energy. The Rumen pH is inversely related to VFA production, which increases with starch content and rate of degradation in the rumen [33, 35]. Consequently, a higher starch degradation rate may lead to a decrease in rumen pH. Among the 3 groups, the DRC goats exhibited the lowest VFA concentrations, accompanied by the correspondingly highest rumen pH. Previous studies [36, 37] have shown an increase in the ruminal propionate concentration and a reduction in the acetate-to-propionate ratio in goats and cows fed diets containing rapidly degradable starch. In the present study, the utilization of SFC tended to increase the propionate concentration and significantly reduce the acetate-to-propionate ratio in the rumen. These findings indicate that steam flaking enhances the rate of cornstarch fermentation in the rumen of goats. In addition, a previous study revealed that the propionate concentration and the propionate-to-acetate ratio in high-gain goats were significantly higher compared to their low-gain counterparts [38]. Acetate production is accompanied by the production of hydrogen, whereas propionate production is accompanied by hydrogen consumption during rumen fermentation [37, 39]. Hydrogen serves as the primary substrate and electron donor for methane synthesis by methanogenic archaea [40]. However, in this study, SFC decreased the acetate-to-propionate ratio, but instead increased GasE. Based on the pathways of carbohydrate fermentation, the hydrogen available for methanogenesis depends not only on the ruminal VFA profiles but also on the amount of dietary carbohydrate fermented [37]. It has been observed that dissolved hydrogen concentrations are greater in the rumens of animals fed readily digestible carbohydrates [40, 41]. In this study, the DRC group demonstrated decreased levels of rumen-degraded starch and exhibited a lower molar concentration of acetate in rumen fluid, which may offer insights into the observed low GasE. The propionate produced in the rumen serves as the primary substrate for hepatic gluconeogenesis and provides energy for overall body metabolism [42]. In the present study, the difference in serum glucose levels was similar to that in the propionate concentration, with increased glucose levels in goats fed SFC compared to those in the DRC group. This result supports the notion that a higher amount of starch in the SFC group underwent ruminal degradation, leading to the production of propionate, which was subsequently absorbed and utilized. Furthermore, our findings align with the results of Rafiee and Darabighane [29], which indicated a decrease in the concentrations of isovalerate and isobutyrate in the SFC group. These ruminal isoacids serve as specific nutrients for cellulolytic bacteria and appear to have a beneficial impact on microbial fermentation [43]. A decrease in isoacids may potentially affect fiber degradation.

Among the carbohydrates degraded in the rumen, approximately 75%–85% of the energy is converted into VFAs, while the remaining energy is lost as CO_2_, CH_4_, and heat [44]. Consequently, rumen fermentation was enhanced in the SFC and DRW groups, but it also led to an increased loss of heat. In the present study, the elevated heat of fermentation in the DRW and SFC groups resulted in increased HI losses, ultimately causing no discernible variation in NE compared to the DRC group. Moreover, the rumen-escaped starch content was higher in the DRC group, suggesting the possibility of partial compensatory starch digestion occurring in the small intestine. Starch digestion is generally more extensive in goats than in cattle fed a similar diet [45]. Therefore, goats may have less restriction of starch digestion in the small intestine than cattle [46]. It has been reported that small intestinal starch digestion can provide approximately 42% more energy than ruminal fermentation [47]. The DRC group may have partially offset the energy loss resulting from reduced rumen fermentation through enhanced small intestinal digestion.

According to the indirect estimation model suggested by Ren et al. [48], the concentration of VFAs in feces represents the process of large intestinal fermentation, while the nutrient content in feces indicates the quantity of undigested nutrients. Based on the fermentation in the large intestine and the remaining nutrient content after fermentation, the nutrient content and digestion of the chyme before reaching the large intestine can be inferred. SFC resulted in a reduction in fecal VFAs concentration and a tendency to increase pH compared to those in the DRC group. Considering the positive correlation between fecal starch content and VFA, as well as the negative correlation with pH [49, 50], the lower fecal VFA concentration and higher pH in the SFC group may indicate reduced hindgut starch fermentation. Furthermore, starch fermentation typically increases the levels of propionate and butyrate [51, 52]. The diminished propionate and butyrate concentrations in the SFC group further indicated a reduction in starch fermentation within the hindgut of goats. Additionally, the DRW group exhibited a decrease in the fecal butyrate concentration. Encouragingly, fecal starch content aligns with the earlier inferences, as the SFC and DRW groups exhibited lower fecal starch contents than the DRC group. These findings suggest enhanced starch digestion in goats fed SFC and DRW before reaching the hindgut. Conversely, the SFC and DRW groups exhibited elevated fecal NDF and ADF levels, indicating a potential negative impact on fiber digestion in rumen. This outcome may be associated with the decline in rumen pH [53].

## Conclusion

This study systematically determined the NE values of the DRC, DRW and SFC in goats. These results provide basic data for formulating accurate feed formula. Compared to those in DRC, the use of DRW and SFC, which are rapidly fermentable starch sources, enhanced the digestibility of starch and the DE of the test diets and ingredients by improving rumen fermentation. However, this improvement also led to increased HI and methane losses from fermentation, thus failing to improve NE. Therefore, reducing dietary heat production and methane emissions from rumen fermentation while increasing digestibility may be the key to improving NE efficiency. Given the unique starch digestion capability of dairy goats, more extensive processing of cereal feeds in high-starch diets does not improve energy efficiency.

## Supplementary Information

Additional file 1. Fig. S1 Effects of different diets on the molar concentrations of acetate and propionate in rumen fluid. *DRC* Dry-rolled corn, *DRW* Dry-rolled wheat, *SFC* Steam-flaked corn. Time = hours after morning feeding. ^a,b^Means without a common superscript are significantly different from each other at *P* < 0.05. *n* = 6.

## Data Availability

All the data generated or analyzed in this study are included in this paper.

## Abbreviations

ADF: Acid detergent fiber
ALB: Albumin
ATTP: Apparent total tract digestibility
CHO: Total cholesterol
CP: Crude protein
DE: Digestible energy
DM: Dry matter
DMI: Dry matter intake
DRC: Dry-rolled corn
DRW: Dry-rolled wheat
FE: Fecal energy
FHP: Fasting heat production
GasE: Gaseous energy
GE: Gross energy
GLU: Glucose
HI: Heat increment
HP: Heat production
ME: Metabolizable energy
NDF: Neutral detergent fiber
NE: Net energy
NEm: Net energy for maintenance
NH_3_-N: Ammonia-nitrogen
OM: Organic material
RE: Retained energy
SEM: Standard error of the mean
SFC: Steam-flaked corn
TG: Triglyceride
TP: Total protein
UE: Urinary energy
UN: Urinary nitrogen
VFA: Volatile fatty acid
