# Supplementary figures and images for: Net energy of grains for dairy goats differed with processing methods and grain types
Source: J Anim Sci Biotechnol. 2025 Jan 5;16:3. doi: 10.1186/s40104-024-01136-y (PMC11700460; doi:10.1186/s40104-024-01136-y)

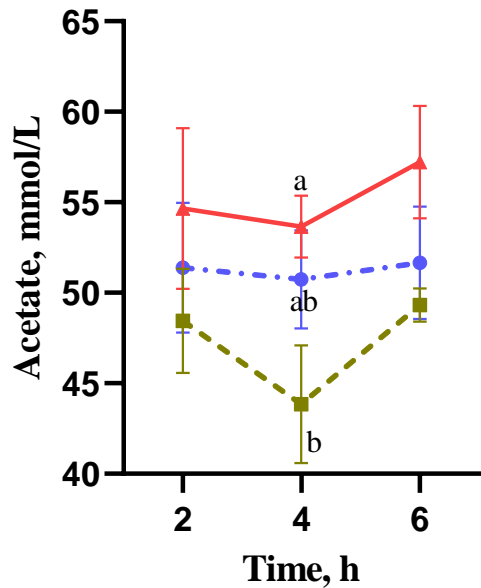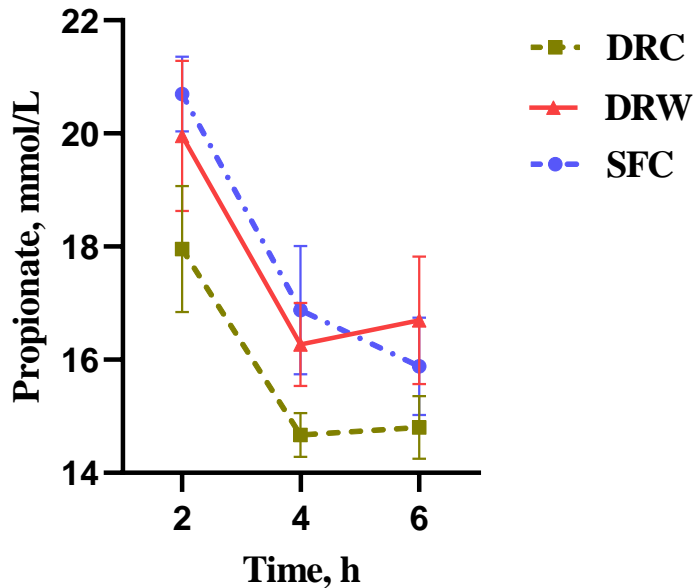

Supplement: Supplementary file 1 — Additional file 1. Fig. S1 Effects of different diets on the molar concentrations of acetate and propionate in rumen fluid. DRC Dry-rolled corn, DRW Dry-rolled wheat, SFC Steam-flaked corn. Time = hours after morning feeding. a,bMeans without a common superscript are significantly different from each other at P < 0.05. n = 6. [file 40104_2024_1136_MOESM1_ESM.pdf]
